# Nomogram Development for Assessing Oncotype DX Recurrence Scores in Breast Cancer: A Chinese Population Study

**DOI:** 10.1002/cam4.70818

**Published:** 2025-03-21

**Authors:** Jiayin Song, Lin Yang, Zhengqi Feng, Liyu Jiang

**Affiliations:** ^1^ Department of Breast Surgery, General Surgery Qilu Hospital, Cheeloo College of Medicine, Shandong University Jinan Shandong China; ^2^ Department of Breast Surgery, General Surgery Qilu Hospital of Shandong University Dezhou Hospital Dezhou China

**Keywords:** breast cancer, nomogram, Oncotype DX, prediction, recurrence

## Abstract

**Background:**

Breast cancer (BC) is the most prevalent cancer among women worldwide, with increasing incidence rates, particularly in China. Given the high costs of Oncotype DX (ODX) testing, which predicts recurrence scores (RSs) on the basis of gene expression, developing a nomogram utilizing clinicopathological variables may provide an accessible alternative for risk stratification.

**Methods:**

We conducted a retrospective analysis of 703 estrogen receptor (ER)‐positive, HER2‐negative T1‐3N0M0 BC patients who underwent ODX testing at Qilu Hospital. A nomogram was developed using multivariate logistic regression to predict low and high RSs in the group. Model performance was validated by receiver operating characteristic curve, calibration curve, and decision curve analysis.

**Results:**

Multivariate analysis revealed that older age, lower histologic grade, a higher ER expression level, a higher proportion of cells expressing progesterone receptor, and a lower proportion of cells expressing Ki‐67 were significantly associated with a patient being in the low‐risk subgroup. A nomogram was then developed using these variables to predict the RS, with an area under the curve (AUC) of 0.811 (95% confidence interval [CI] = 0.772–0.850) in the development group and 0.794 (95% CI = 0.737–0.851) in the validation group. Calibration and decision curve analyses further confirmed the nomogram's clinical utility. Moreover, a comparison between the TAILORx‐nomogram and our nomogram was conducted, which proved that our nomogram has better predictive accuracy and reliability in Chinese BC patients.

**Conclusion:**

We present the first nomogram for predicting the RS in Chinese patients with BC on the basis of clinicopathological factors. This model could aid in identifying patients who may not need ODX testing and serve as a cost‐effective alternative for those unable to access ODX, thereby optimizing treatment decisions and enhancing patient management in resource‐limited settings.

## 1 Introduction

Breast cancer (BC) is the most prevalent cancer among women worldwide and is the second leading cause of mortality for women overall [1]. According to a recent report from the International Agency for Research on Cancer (IARC), approximately 2.26 million women were diagnosed with BC globally in 2020, accounting for 11.7% of all cancer diagnoses, and BC has surpassed lung cancer as the most prevalent cancer [2]. In China, the incidence of BC has been increasing steadily [3]. It has been reported that 416,371 Chinese women were diagnosed with BC in 2020, accounting for 18.4% of all new cancer cases in China, and that all BC‐related deaths in China accounted for 17.1% of deaths globally in 2020 [3, 4], which places significant strain on the health care systems of China.

As one of the most important systematic treatments, chemotherapy [5], which can be given after surgery to reduce the risk of recurrence or to palliatively treat unresectable metastatic tumors, has been shown to improve the prognosis of patients with BC [6, 7]. A study based on a real‐world population reported that adjuvant chemotherapy could improve 5‐year overall survival (OS) rates from 87.6% to 92.1% [8]. Although chemotherapy can play a positive role in patients with BC, the response to such treatment varies significantly among individuals [9, 10]. Moreover, adverse effects of chemotherapy, such as hair loss, fatigue, nausea, vomiting, immunosuppression, and drug‐induced interstitial pneumonitis, can sometimes outweigh the benefits of treatment for some patients [11]. Therefore, finding an accurate assessment method to predict the risk of recurrence and the need for chemotherapy in BC patients is highly important.

With improvements in genetic diagnostic technologies, clinicians are now able to assess the prognosis, risk of recurrence, and treatment response of BC patients by analyzing the expression levels of specific genes in tumor tissues [12], which has significantly propelled the development of precision medicine, enabling more tailored and effective therapeutic strategies for individual patients [13, 14]. For example, the 70‐gene expression signature Mammaprint was the first fully commercialized multivariate predictive test utilizing a microarray‐based multigene assay to assess the risk of relapse in newly diagnosed BC patients [15]. Oncotype DX (Genomic Health, Redwood City, CA; hereafter referred to as ODX) is a genetic test that calculates a recurrence score (RS) on the basis of the RNA expression levels of 21 genes in tumor tissue from patients with invasive BC, including 16 tumor‐related genes and five reference genes, and is one of the most commonly used genetic diagnostic technologies in the clinic to predict the outcome and response to chemotherapy of hormone receptor (HR)‐positive and human epidermal growth factor receptor 2 (HER2)‐negative BC [16, 17, 18]. Previous studies reported that chemotherapy had no significant effect on the ODX low‐risk group but improved the survival of patients in the high‐risk group [19, 20].

Although numerous studies have demonstrated that ODX is a valuable tool for identifying individuals who could benefit from chemotherapy, and it has also been recommended by several guidelines, such as the American Society of Clinical Oncology (ASCO) and the National Comprehensive Cancer Network (NCCN) [21, 22], the cost of the test is high (approximately 5000 RMB), limiting its availability in China and causing some patients to choose chemotherapy directly [23]. Previous studies have reported that nomograms to predict low (0–25) and high (25–100) RS groups on the basis of the clinicopathological variables of patients are practicable [24, 25]. While most of these studies were conducted in Western populations, nomograms based on Asian populations, especially the Chinese population, are rare. In this study, we performed a retrospective analysis of 703 patients who underwent ODX at our hospital to develop a nomogram as a surrogate prediction model for high‐risk or low‐risk ODX RS test results. We found that patient age, histologic grade, ER expression, proportion of cells expressing progesterone receptor (PR), and proportion of cells expressing Ki‐67 of tumor tissues were suitable predictive variables for nomogram development. Internal validation was then performed to assess the model's performance, followed by external validation using an independent cohort to further confirm its predictive accuracy and reliability.

## 2 Materials and Methods

### 2.1 Patient and Pathology Variables Selection

We retrospectively analyzed 703 patients with estrogen receptor (ER)‐positive, HER2‐negative, T1‐3N0M0 BC (UICC TNM 8th Edition) who underwent surgery and ODX testing at Qilu Hospital of Shandong University between January 2020 and May 2024. Patients who received neoadjuvant chemotherapy were excluded. Informed consent for ODX was obtained from all participants.

Clinicopathological data, including patient age, histologic subtype, histologic grade, accompaniment of DCIS, size, T stage, ER status, PR status, ER expression, PR expression, proportion of cells expressing ER, proportion of cells expressing PR, HER2 status, proportion of cells expressing Ki‐67, and the RS results of tumor tissues, were obtained from electronic medical records. Staging for all tumors was conducted in accordance with the “TNM Classification of Malignant Tumors” (8th Edition). The ER status, ER expression, proportion of cells expressing ER, PR status, proportion of cells expressing PR and proportion of cells expressing Ki‐67 were evaluated by three independent experienced pathologists on the basis of the percentage of staining intensity (intensity score) and positive cells (proportion score) assessed using immunohistochemistry (IHC) staining. HER2 status was evaluated by IHC or fluorescence in situ hybridization (FISH) assays.

### 2.2 Statistical Analysis

In this study, patients were divided into low (RS, 0–25) and high (RS, 26–100) groups on the basis of the RS threshold that was used in clinical trials such as TAILORx and RxPONDER [19, 26]. The chi‐squared test and Fisher's exact test were used to compare the RS results between clinicopathological characteristics. Univariate and stepwise multiple logistic regression were used to filter potential valuable variables (*p* < 0.05) in the development group, which included 465 patients. Nomograms for low and high RSs were then developed using variables that yielded significant results in the multivariate logistic regression analysis. The receiver operating characteristic (ROC) curve was then plotted, and the area under the curve (AUC), accuracy (ACC), true positive rate (TPR), true negative rate (TNR), positive predictive value (PPV), and negative predictive value (NPV) were further calculated to determine the performance of the nomogram. Additional calibration was conducted to evaluate the discrepancies between the actual RS and the predictions generated by the nomogram. Decision curve analysis (DCA) was further used to evaluate the clinical benefits and utility of the nomogram. The ROC curve, calibration, and DCA were further conducted in an independent validation group containing 238 BC patients to validate the performance of our model. Moreover, the *C*‐index and Brier score were applied to evaluate the calibration of the models, and the net reclassification index (NRI) and integrated discrimination improvement (IDI) were also used to evaluate the nomogram's clinical utility. The data analyses were performed via the R statistical package ver. 4.4.1 (http://r‐project.org/). The significance level was set at 0.05, and all *p* values reported were two‐sided.

## 3 Results

### 3.1 Patient Characteristics

A total of 703 patients with ER‐positive, HER2‐negative, T1‐3N0M0 BC were included in this study, among which 451 patients had low‐risk (RS, 0–25) and 252 patients had high‐risk (RS, 26–100) BC. Compared with those in the low‐risk group, patients in the high‐risk group were significantly younger (*p* < 0.001), had a higher histologic grade (*p* < 0.001), and a higher proportion of cells expressing Ki‐67 (*p* < 0.001), whereas the ER expression (*p* < 0.001), proportion of cells expressing ER (*p* < 0.001), PR status (*p* = 0.017), PR expression (*p* < 0.001), and proportion of cells expressing PR (*p* < 0.001) were markedly lower. With respect to the histologic subtype (*p* = 0.399), DCIS status (*p* = 0.543), size (*p* = 0.997), T stage (*p* = 0.120), and HER2 status (*p* = 0.297) of the tumors, there was no significant difference between the groups. Among the 703 patients, 465 (66.1%) patients were included in the development group, and 238 (33.9%) patients were included in the validation group. The patient characteristics of the total cohort, development group, and validation group are shown in Table 1.

**TABLE 1:** Baseline characteristics and association between dichotomized Oncotype DX score and clinicopathologic variables.

	Total cohort			Development group			Validation group		
	RS 0–25 (*N* = 451)	RS 26–100 (*N* = 252)	*p*	RS 0–25 (*N* = 293)	RS 26–100 (*N* = 172)	*p*	RS 0–25 (*N* = 158)	RS 26–100 (*N* = 80)	*p*
Age (mean ± SD)	53.9 ± 9.9	48.9 ± 8.8	< 0.001	54.1 ± 9.7	49.2 ± 9.0	< 0.001	53.4 ± 10.5	48.1 ± 8.4	< 0.001
Histologic subtypes									
IDC	413 (91.6%)	236 (93.7%)	0.399	274 (93.5%)	162 (94.2%)	0.928	139 (88%)	74 (92.5%)	0.394
Others	38 (8.4%)	16 (6.3%)		19 (6.5%)	10 (5.8%)		19 (12%)	6 (7.5%)	
Histologic grade									
1	61 (13.5%)	19 (7.5%)	< 0.001	39 (13.3%)	11 (6.4%)	< 0.001	22 (13.9%)	8 (10%)	0.219
2	376 (83.4%)	203 (80.6%)		246 (84%)	138 (80.2%)		130 (82.3%)	65 (81.2%)	
3	14 (3.1%)	30 (11.9%)		8 (2.7%)	23 (13.4%)		6 (3.8%)	7 (8.8%)	
Accompanied by DCIS									
No	120 (26.6%)	61 (24.2%)	0.543	76 (25.9%)	39 (22.7%)	0.499	44 (27.8%)	22 (27.5%)	1
Yes	331 (73.4%)	191 (75.8%)		217 (74.1%)	133 (77.3%)		114 (72.2%)	58 (72.5%)	
Size (mean ± SD)	1.7 ± 1.3	1.8 ± 0.7	0.887	1.8 ± 1.4	1.8 ± 0.7	0.636	1.6 ± 0.7	1.8 ± 0.8	0.258
T stage									
1	364 (80.7%)	190 (75.4%)	0.120	230 (78.5%)	132 (76.7%)	0.746	134 (84.8%)	58 (72.5%)	0.036
2	87 (19.3%)	62 (24.6%)		63 (21.5%)	40 (23.3%)		24 (15.2%)	22 (27.5%)	
ER intensity									
1	205 (45.5%)	180 (71.4%)	< 0.001	138 (47.1%)	120 (69.8%)	< 0.001	67 (42.4%)	60 (75%)	< 0.001
2	246 (54.5%)	72 (28.6%)		155 (52.9%)	52 (30.2%)		91 (57.6%)	20 (25%)	
ER proportion (mean ± SD)	86.2 ± 10.4	80.7 ± 14.4	< 0.001	85.9 ± 11.2	80.7 ± 14.6	< 0.001	86.7 ± 8.9	80.8 ± 14.2	< 0.001
PR status									
Negative	17 (3.8%)	21 (8.3%)	0.017	12 (4.1%)	16 (9.3%)	0.038	5 (3.2%)	5 (6.2%)	0.436
Positive	434 (96.2%)	231 (91.7%)		281 (95.9%)	156 (90.7%)		153 (96.8%)	75 (93.8%)	
PR intensity									
0	16 (3.5%)	21 (8.3%)	< 0.001	11 (3.8%)	16 (9.3%)	0.01	5 (3.2%)	5 (6.2%)	0.009
1	170 (37.7%)	119 (47.2%)		107 (36.5%)	73 (42.4%)		63 (39.9%)	46 (57.5%)	
2	265 (58.8%)	112 (44.4%)		175 (59.7%)	83 (48.3%)		90 (57%)	29 (36.2%)	
PR proportion (mean ± SD)	70.1 ± 28.4	56.9 ± 33.6	< 0.001	69.9 ± 29.2	55.9 ± 33.9	< 0.001	70.5 ± 26.9	59.2 ± 33.1	0.009
HER‐2 status									
0	181 (40.1%)	116 (46%)	0.297	113 (38.6%)	77 (44.8%)	0.279	68 (43%)	39 (48.8%)	0.538
1	186 (41.2%)	96 (38.1%)		123 (42%)	70 (40.7%)		63 (39.9%)	26 (32.5%)	
2N	84 (18.6%)	40 (15.9%)		57 (19.5%)	25 (14.5%)		27 (17.1%)	15 (18.8%)	
Ki‐67 proportion (mean ± SD)	15.3 ± 9.6	21.9 ± 13.8	< 0.001	15.2 ± 9.8	22.6 ± 14.5	< 0.001	15.5 ± 9.5	20.3 ± 12.1	0.002

### 3.2 Development of the Nomogram

To filter potential valuable variables that were correlated with the RS results, univariate logistic regression analysis was first conducted in the development group. As shown in Table 2, our univariate results revealed that age, histologic grade, ER expression, proportion of cells expressing ER, PR status, proportion of cells expressing PR, and proportion of cells expressing Ki‐67 were potential variables that influenced the results of the RS. To further screen the above variables, stepwise multiple logistic regression was then performed, which revealed that older age (OR = 1.091), higher ER expression (OR = 3.524), and higher proportion of cells expressing PR (OR = 1.027) were positively correlated with low RS in BC patients, whereas higher histologic grade (OR = 0.108) and higher proportion of cells expressing Ki‐67 (OR = 0.942) were negative predictors of low RS results. The odds ratios, 95% confidence intervals (CIs), and *p* values for the RS and each clinicopathological variable are shown in Table 2.

**TABLE 2:** Univariate and multivariate logistic regression analysis for each clinicopathological factor to predict recurrence score in the model development group.

	Univariate analysis		Multivariate analysis	
	OR (95% CI)	*p*	OR (95% CI)	*p*
Age (mean ± SD)	**1.056 (1.025–1.079)**	**< 0.001**	**1.091 (1.060–1.124)**	**< 0.001**
Histologic subtypes				
IDC	Ref	Ref		
Others	1.123 (0.520–2.570)	0.773		
Histologic grade				
1	Ref	Ref	Ref	Ref
2	0.503 (0.239–0.981)	0.054	0.571 (0.248–1.229)	0.166
3	**0.098 (0.033–0.268)**	**< 0.001**	**0.108 (0.279–0.387)**	**0.001**
Accompanied by DCIS				
No	Ref	Ref		
Yes	0.837 (0.534–1.297)	0.431		
Size (mean ± SD)	1.033 (0.887–1.230)	0.686		
T stage				
1	Ref	Ref		
2	0.904 (0.578–1.425)	0.660		
ER intensity				
1	Ref	Ref	Ref	Ref
2	**2.592 (1.748–3.880)**	**< 0.001**	**3.524 (2.072–6.131)**	**< 0.001**
ER proportion (mean ± SD)	**1.033 (1.017–1.049)**	**< 0.001**	1.008 (0.990–1.028)	0.382
PR status				
Negative	Ref	Ref	Ref	Ref
Positive	**2.402 (1.113–5.316)**	**0.026**	—	—
PR intensity				
0	Ref	Ref	Ref	Ref
1	2.132 (0.944–4.977)	0.072	—	—
2	**3.067 (1.375–7.077)**	**0.007**	—	—
PR proportion (mean ± SD)	**1.014 (1.008–1.020)**	**< 0.001**	**1.027 (1.017–1.037)**	**< 0.001**
HER‐2 status				
0	Ref	Ref		
1	1.197 (0.793–1.810)	0.392		
2N	1.554 (0.902–2.730)	0.118		
Ki‐67 proportion (mean ± SD)	**0.949 (0.933–0.965)**	**< 0.001**	**0.942 (0.921–0.962)**	**< 0.001**

*Note:* Bold values indicates statistically significant value.

**FIGURE 1:**
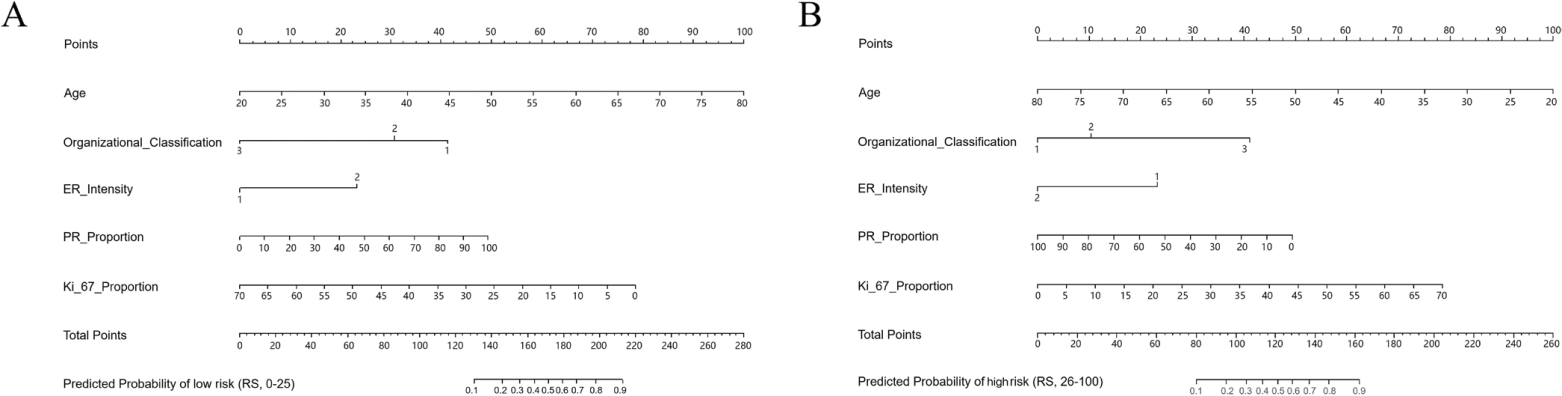
Nomogram to predict the probability of a low recurrence score (A) and high recurrence score (B). The nomogram was developed using variables that were significant in the multivariate logistic regression analysis.

On the basis of these results, the five significant variables of the explanatory model were used to develop a nomogram to predict the low risk (Figure 1A) and high risk (Figure 1B) RSs of ODX. In summary, each of the five predicting clinicopathological variables was assigned a point value by aligning the variable's numerical line with the topmost “point” line. The points for all five variables were then summed, and the total points were further aligned with the “predicted probability” numerical line, allotting the final predicted probability for a low risk or a high risk for an individual patient.

### 3.3 Evaluating the Performance of the Nomogram Model

**FIGURE 2:**
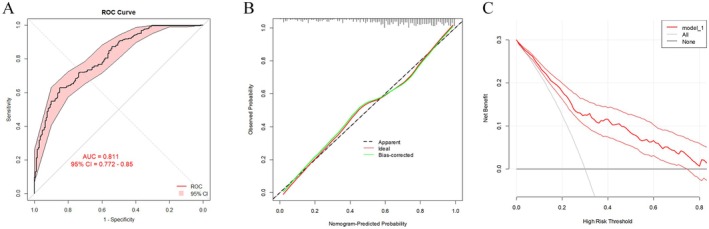
Receiver operating characteristic curves of the nomogram (A), calibration plot (B), and decision curve analysis (C) in the model development group.

**FIGURE 3:**
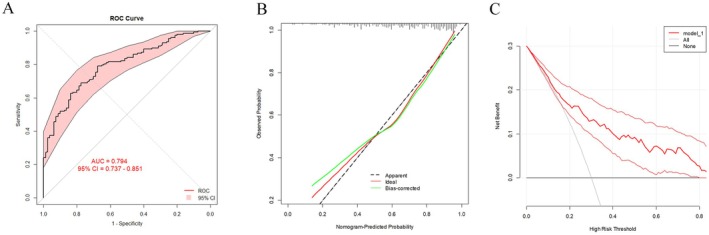
Receiver operating characteristic curves of the nomogram (A), calibration plot (B), and decision curve analysis (C) in the model validation group.

After the development of the nomogram model, we further plotted an ROC curve to evaluate its discriminatory efficiency in the model development group. As shown in Figure 2A, the AUC of the developed model was 0.811 (95% CI = 0.772–0.850). Moreover, calibration analysis was then conducted to evaluate any discrepancies between the actual RS and the values predicted by the nomogram (Figure 2B). DCA was also conducted, which indicated that our nomogram model had significant utility for clinical decision‐making (Figure 2C). To further validate the accuracy and specificity of our nomogram model, the model was then adapted to the validation group to predict the probability of a low RS. As shown in Figure 3A, the AUC was 0.794 (95% CI = 0.737–0.851), demonstrating a discriminative ability comparable to that of the nomogram development group. The calibration and DCA results of the model validation group are shown in Figure 3B,C.

### 3.4 Comparison of a Western Nomogram With the Chinese Nomogram

**FIGURE 4:**
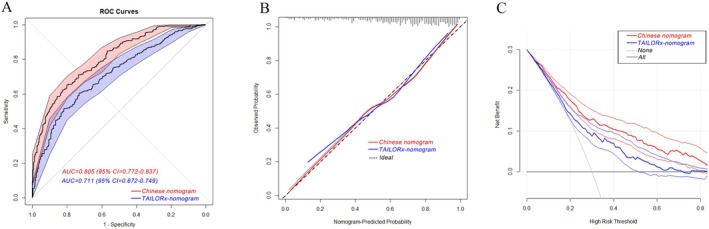
Receiver operating characteristic curve of the nomogram (A), calibration plot (B), and decision curve analysis (C) in Chinese breast cancer patients. The red curve indicates our nomogram model, and the blue curve indicates the TAILORx‐nomogram model.

A nomogram prediction model for ODX has been built on the basis of Western populations. For example, a study conducted at the University of Tennessee Medical Center using a large dataset from the National Cancer Database (NCDB) reported the use of a nomogram for predicting ODX scores with clinicopathologic data, which involved factors such as age, size, grade, progesterone, and histology [24]. To verify whether the nomogram built in our study is more suitable for Chinese BC patients than the TAILORx‐nomogram is, the patient characteristics of the TAILORx‐nomogram and our nomogram were used to calculate ROC curves for Chinese BC patients. As shown in Figure 4A, the AUC was 0.711 (95% CI = 0.672–0.749) for the TAILORx‐nomogram and 0.805 (95% CI = 0.772–0.837) for our nomogram, and the difference was highly significant according to the Delong test. The sensitivity and specificity of the ROCs of the Chinese nomogram and the TAILORx‐nomogram are presented in Table 3. Moreover, calibration and DCA were also conducted, and the *C*‐index and Brier score of the calibration curves together with the NRI and IDI of the DCA curves were also determined to compare the calibration and clinical benefits of the nomograms, which further proved that our nomogram model was more suitable for predicting ODX results in Chinese patients with BC than the TAILORx‐nomogram (Table 4).

**TABLE 3:** Sensitivity/specificity analyses of ROC models for Chinese nomogram and TAILORx nomogram.

	TAILORx‐nomogram	Chinese nomogram	*z‐statistic*	*p*
AUC	0.711 (0.672–0.749)	0.805 (0.772–0.837)	−5.715	< 0.001
ACC	0.650 (0.607–0.693)	0.718 (0.677–0.755)		
TPR	0.605 (0.528–0.719)	0.710 (0.647–0.769)		
TNR	0.730 (0.578–0.785)	0.734 (0.642–0.790)		
PPV	0.801 (0.750–0.821)	0.827 (0.789–0.850)		
NPV	0.508 (0.464–0.549)	0.585 (0.536–0.633)		

**TABLE 4:** Performance metrics of calibration curves and DCA curves in different nomogram models.

	TAILORx‐nomogram	Chinese nomogram	*p*
Calibration curves			
*C*‐index	0.711 (0.672–0.749)	0.805 (0.772–0.837)	< 0.001
Brier score	0.201 (0.185‐0.212)	0.168 (0.151‐0.180)	< 0.001
DCA curves			
NRI	Reference	0.459 (0.445–0.777)	< 0.001
IDI	Reference	0.107 (0.063–0.142)	< 0.001

## 4 Discussion

Chemotherapy resistance is one of the most significant factors contributing to recurrence and mortality in BC patients [7]. Therefore, identifying patients who are resistant or sensitive to chemotherapy is highly important, especially given the potential adverse effects of chemotherapy [27]. ODX testing, also known as the 21‐gene assay, is the most widely used BC genomic assay in the world; it is supported by the American Society of Clinical Oncology and the National Comprehensive Cancer Network for decision‐making about the treatment of node‐negative, ER‐positive BC patients and is accepted as a clinically valuable test [28, 29, 30]. Although the benefit of ODX testing has been proven by numerous studies [31, 32], its high cost has limited its application. It has been reported that only approximately one‐third of BC patients in the USA and 20% of those in Europe have undergone ODX testing [33]. The ODX test is a heavy financial burden for BC patients. Zheng et al. reported that the diagnostic cost of each assay is currently around 5000 RMB in China [23]. Given the high cost of the ODX test, developing nomograms to identify patient groups needing chemotherapy at a lower cost is highly important. Such tools can help select appropriate treatments for patients who cannot afford ODX testing.

Some nomograms to predict the ODX results have been studied in different countries. For example, a research team at Tennessee University developed a nomogram using NCDB database data, identifying age, tumor size, grade, PR status, and BC histologic type as strong predictors of ODX results. This model demonstrated high accuracy in distinguishing between low (≤ 25) and high (> 25) RSs, achieving a C‐index of 0.81 [24]. However, a Korean study reported that the nomogram developed at Tennessee University lacks accuracy in predicting RSs for Asian populations. This highlights the need to develop nomograms based on datasets encompassing diverse racial groups for more accurate predictions [34].

Since previous studies have indicated that the clinicopathologic characteristics of patients could be used more cost‐effectively to predict ODX test results [35], the correlations between clinicopathologic characteristics and ODX results were further analyzed in Chinese BC patients. We found that age, grade, ER expression, the proportion of cells expressing PR, and the proportion of cells expressing Ki‐67 were potential predictors of ODX results for Chinese BC patients, and a nomogram model predicting the results of ODX was built on the basis of the above factors. These five clinicopathologic variables can be collected through any pathologic examination and are clinically established variables for predicting outcomes, and therefore, they are suitable for clinical usage. Among the five factors, PR and Ki‐67 are target genes in the ODX assay, and their correlation with ODX results has been widely emphasized. In a single‐center study, PR negativity was associated with a notably higher RS than PR positivity was, suggesting that PR status may be a useful predictor of the RS [36]. Lee et al. reported that a higher Ki‐67 level was associated with higher RS scores [33]. For age, grade, and ER, the correlations between these factors and RS results have also been proven, which indicates that older age, lower grade, and positive ER status are predictors of a low RS [24, 33, 37]. ROC curve, calibration curve, and DCAs were then conducted to examine the sensitivity, specificity, and clinical significance of the nomogram model, which further confirmed that the combination of age, grade, ER expression, proportion of cells expressing PR, and proportion of cells expressing Ki‐67 were useful predictors of the RS in the Chinese population with BC.

Previous reports have highlighted that the nomogram developed at Tennessee University, although effective in Western populations, has shown limited accuracy in predicting RSs for Asian BC patients [34]. Recognizing this limitation, we undertook a comparative analysis of our Chinese‐specific nomogram model against the TAILORx‐nomogram model [24], with a focus on enhancing the predictive accuracy for the unique characteristics of the Chinese population. Unlike the TAILORx‐nomogram, our nomogram did not include the size or histology of the tumors, while the ER expression and proportion of cells expressing Ki‐67 were taken into account for ODX result prediction. Our findings revealed that, compared with the TAILORx nomogram, our nomogram demonstrated superior accuracy in predicting the RS for Chinese patients with BC, confirming the enhanced clinical utility of a model tailored to local population characteristics. These results support the importance of developing region‐specific predictive tools to inform more personalized treatment strategies and optimize clinical outcomes.

In conclusion, we developed a nomogram that predicts the RS results of ODX on the basis of factors such as age, grade, ER expression, proportion of cells expressing PR, and proportion of cells expressing Ki‐67. This model is the first of its kind and is based on data from Chinese BC patients. Our nomogram has the potential to serve as a valuable tool for identifying patients who may or may not require further ODX testing. Additionally, it could provide an alternative for patients who are unable to afford the ODX test or for whom it is not readily available.

## Author Contributions

**Jiayin Song:** data curation (equal), investigation (equal), methodology (equal), formal analysis (equal); writing – original draft (equal). **Lin Yang:** conceptualization (equal), data curation (equal), formal analysis (equal). **Zhengqi Feng:** software (equal), writing – review and editing (equal). **Liyu Jiang:** supervision (equal), writing – original draft (equal), writing – review and editing (equal).

## Ethics Statement

This project was reviewed and approved by the Ethics Committee of Qilu Hospital (KYLL‐2024(ZM)‐436).

## Conflicts of Interest

The authors declare no conflicts of interest.

## Data Availability

The datasets generated and/or analyzed during the current study are available from the corresponding author upon reasonable request.
